# Supplementary material for: DMRforPairs: identifying Differentially Methylated Regions between unique samples using array based methylation profiles
Source: BMC Bioinformatics. 2014 May 15;15:141. doi: 10.1186/1471-2105-15-141 (PMC4046028; doi:10.1186/1471-2105-15-141)
Supplement: Additional file 2 — DMRforPairs output for the comparison of A431-MCF7 and NA17018-NA17105. Please start from the HTML files in each folder. Available via the BMC Bioinformatics website. [file 1471-2105-15-141-S2.zip › 1394847754114233_MOESM2_ESM/A431_MCF7/figures/11124.pdf]

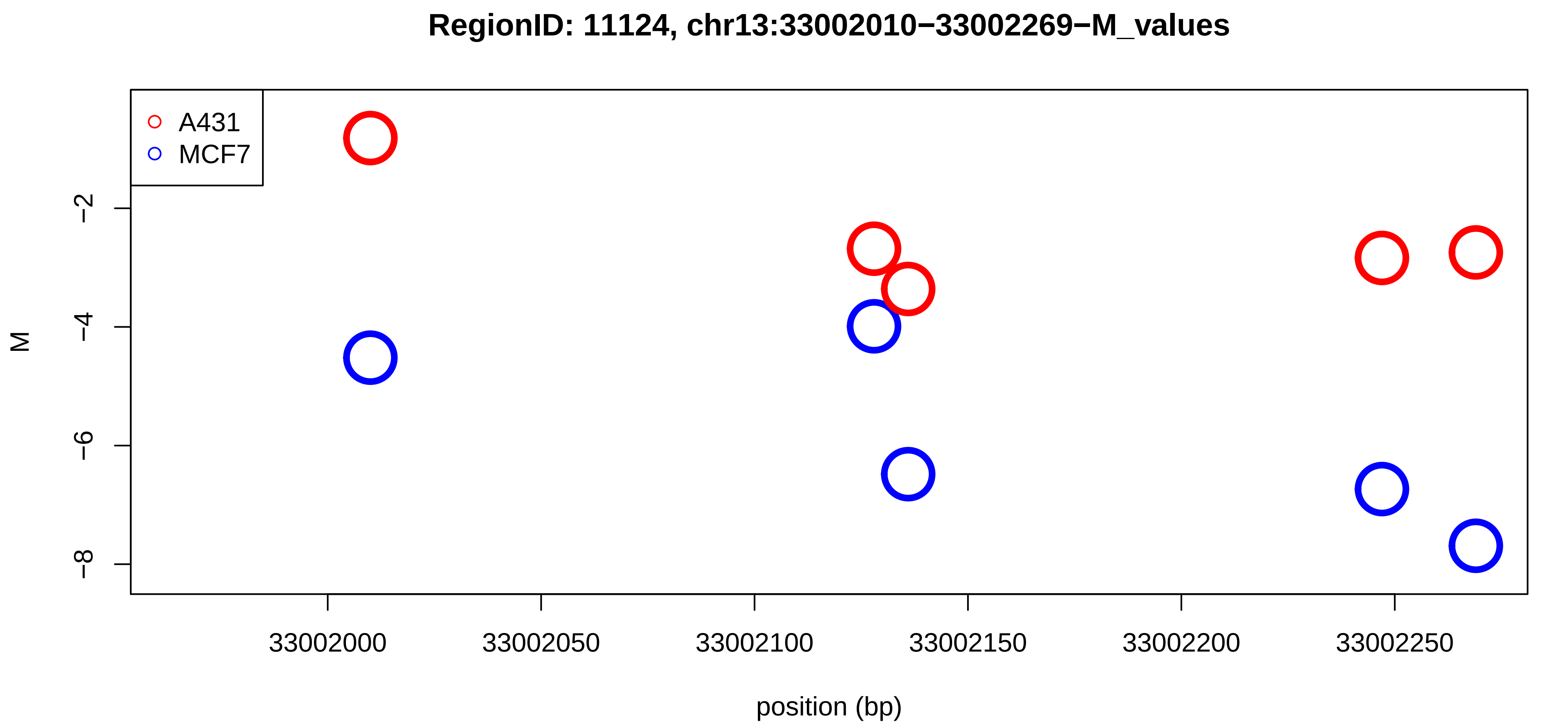
RegionID: 11124, chr13:33002010−33002269−M_values
A431
MCF7
M
−2
−4
−6
−8
33002000
33002050
33002100
33002150
33002200
33002250
position (bp)

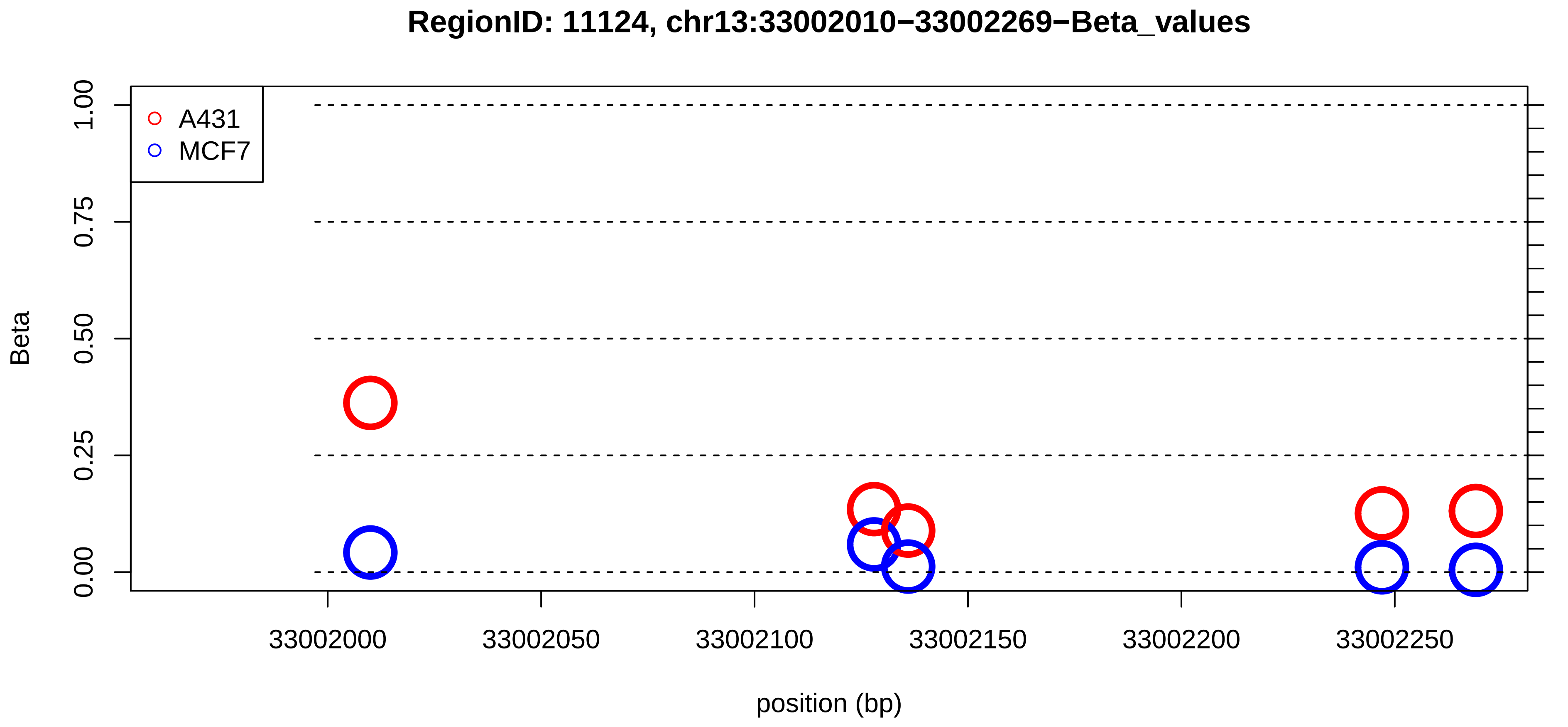
RegionID: 11124, chr13:33002010−33002269−Beta_values
A431
MCF7
Beta
1.00
0.75
0.50
0.25
0.00
33002000
33002050
33002100
33002150
33002200
33002250
position (bp)

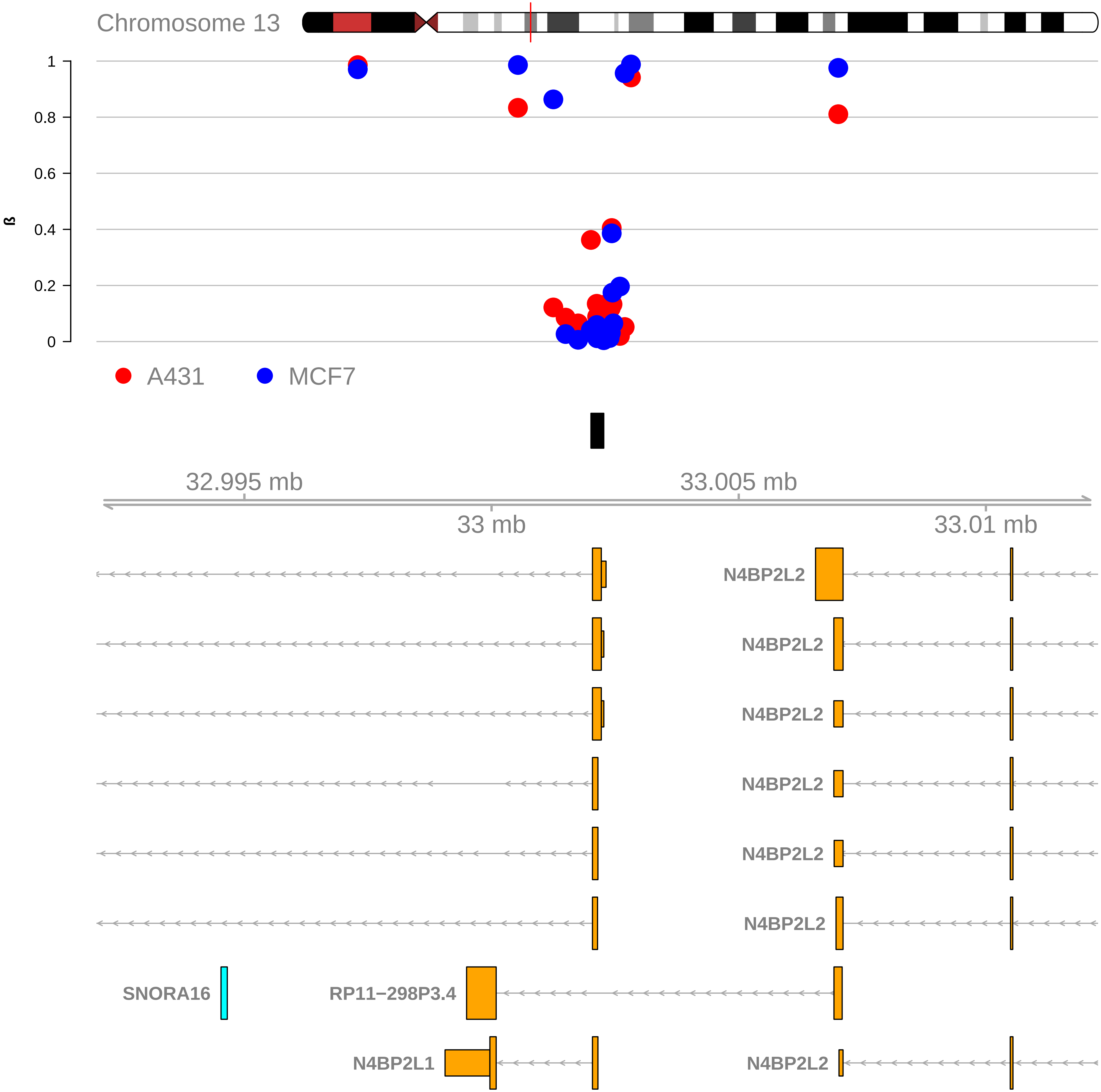

Chromosome 13
ß
1
0.8
0.6
0.4
0.2
0
A431
MCF7
32.995 mb
33.005 mb
33 mb
33.01 mb
N4BP2L2
N4BP2L2
N4BP2L2
N4BP2L2
N4BP2L2
N4BP2L2
SNORA16
RP11−298P3.4
N4BP2L1
N4BP2L2
